# Second-order-like cluster-monomer transition within magnetic fluids and its impact upon the magnetic susceptibility

**DOI:** 10.1186/1556-276X-7-167

**Published:** 2012-03-05

**Authors:** Jing Zhong, Qing Xiang, Letícia O Massa, Fanyao Qu, Paulo C Morais, Wenzhong Liu

**Affiliations:** 1 Department of Control Science and Engineering, Huazhong University of Science and Technology, Wuhan, 430074, China; 2 Instituto de Física, Núcleo de Física Aplicada, Universidade de Brasília, Brasília DF, 70910-900, Brazil

**Keywords:** magnetic fluid, inverse initial susceptibility, cluster disruption, critical temperature, Langevin function

## Abstract

The low-field (below 5 Oe) ac and dc magnetic response of a magnetic fluid [MF] sample in the range of 305 to 360 K and 410 to 455 K was experimentally and theoretically investigated. We found a systematic deviation of Curie's law, which predicts a linear temperature dependence of inverse initial susceptibility in the range of our investigation. This finding, as we hypothesized, is due to the onset of a second-order-like cluster-to-monomer transition with a critical exponent which is equal to 0.50. The susceptibility data were well fitted by a modified Langevin function, in which cluster dissociation into monomers, at the critical temperature [T*], was included. In the ac experiments, we found that T* was reducing from 381.8 to 380.4 K as the frequency of the applied field increases from 123 to 173 Hz. In addition, our ac experiments confirm that only monomers respond for the magnetic behavior of the MF sample above T*. Furthermore, our Monte Carlo simulation and analytical results support the hypothesis of a thermal-assisted dissociation of chain-like structures.

**PACS: **75.75.-C; 75.30.Kz; 75.30.Cr.

## Introduction

The interest in magnetic fluids [MFs] has increased enormously in the last decade, particularly due to the opportunities they provide for applications in the medical field [1-7]. Among others, MFs have been used as an excellent material platform for the development of magnetic immunoassay [1,2], contrast agents for magnetic resonance imaging [3,4], and material devices for magnetohyperthermia [5-7]. Deep understanding of magnetic susceptibility, however, is a key issue not only from the fundamental point of view, but also while tailoring nanosized magnetic materials for medical applications [1-7]. The design of nanosized magnetic particles, taking into account the maximization of the materials' response in terms of their use for diagnosis, imaging, and therapy, requires the knowledge of the temperature dependence of the magnetic susceptibility under the action of applied dc and ac fields.

In this context, the widely accepted concept is a linear relationship between the inverse initial magnetic susceptibility (1/*χ*) and the temperature (*T*), which is accounted for by the first-order Langevin function. Nevertheless, unusual deviations of linearity at temperatures within the range of interest for the medical applications, with no conclusive explanation yet, have been reported [8]. Since an interaction among particles in a MF sample, either modulated or not by external fields, cannot be ignored and leads to expontaneous agglomeration in clusters or chain-like structures (dimers, trimers, etc.) [9-11], the superlinear deviation of the 1/*χ *versus *T *curve has been attributed to magnetic dipolar interaction among the nanosized particles [12]. The Langevin function, however, includes no interaction among the suspended particles, and therefore, clusters are ruled out from the classical description. Nevertheless, at high enough temperatures, the phenomenon of thermally assisted cluster disruption within MF samples has been reported [13]. Therefore, to understand the underlying physics of this nonlinearity in magnetization, a more complete physical model is highly demanded. The new model should take into account both monomers and clusters in MFs.

In this study, we report the unusual superlinear deviation in the temperature dependence of the inverse initial magnetic susceptibility in a magnetite-based (Fe_3_O_4_) MF sample in the temperature range of 305 to 360 K. The observed breakdown of Curie's law, which scales linearly the inverse susceptibility with temperature, indicates that besides the usual tendency of alignment of magnetic moments with the applied field, there exists an additional thermally assisted physical process connected to cluster disruption within MF samples. Therefore, we propose a model in which a chain-like disruption at a typical transition temperature (T*) is incorporated. We found that the extended model reproduces quite well the observed superlinear deviation. Additionally, the above-mentioned superlinear deviation of the (1/*χ*) × *T *data for chains of particles is supported by Monte Carlo [MC] simulation and herein incorporated.

## Experimental description

In order to verify the universality of the experiment regarding the relationship of inverse initial susceptibility and temperature, we explored the experiments under dc and ac magnetic fields, the latter at different frequencies. The MF sample used in our experiment was a commercial magnetic colloid (EFH1, Ferrotec Corporation, Santa Clara, CA, USA), consisting of Fe_3_O_4 _magnetite nanoparticles (mean particle diameter of 10 nm) suspended in light mineral oil. The applied magnetic fields were 5 and 2 Oe (amplitude) for dc and ac experiments, respectively. The ac experiments were performed at 123 and 173 Hz.

**Figure 1:**
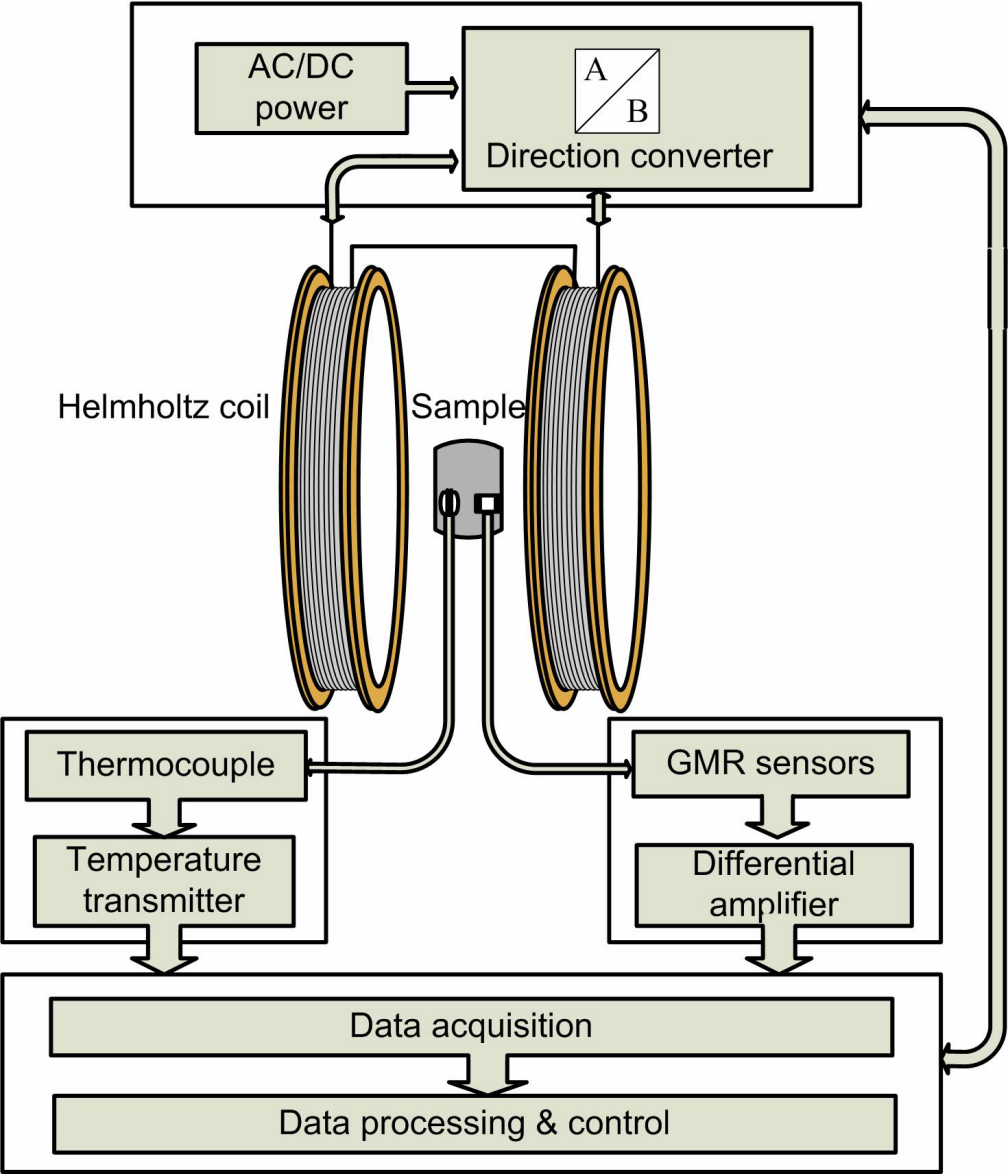
**Schematic diagram of the measurement system suitable for dc/ac initial magnetic susceptibility detection**.

The chord magnetic susceptibility (*χ *= *M*/*H*) can be obtained by measuring the sample's magnetization (*M*) induced by a weak external dc/ac field (*H*). The homemade experimental system designed to measure the MF sample's initial susceptibility is schematically shown in Figure 1. The Helmholtz coils were driven by a dc/ac power supply to generate a uniform dc/ac excitation field at the sample's position; the direction of the dc applied field can be altered by a relay. The temperature of the MF sample was measured using a thermocouple (Pt100) and converted using a temperature transmitter (RS1852290). A weak magnetization signal collected from the MF sample was detected using the two-axis giant magnetoresistive sensors (HMC1022) with an amplification circuit. Both temperature and magnetization data of the sample were acquired through a data acquisition card (PCI-6251, National Instruments, Austin, TX, USA). LabVIEW was used as a human-computer interface for observation and control.

**Figure 2:**
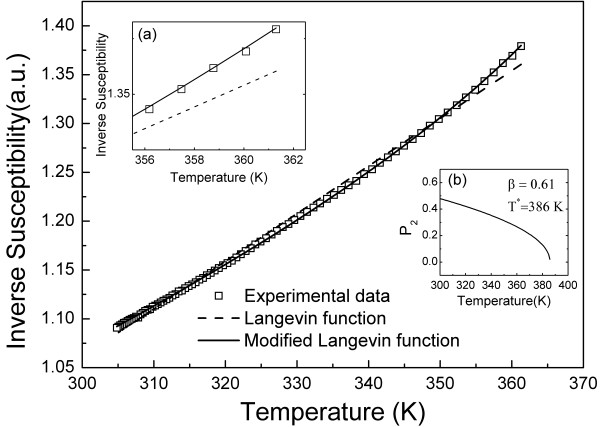
**Experimental data and the fitting curves using the Langevin function**. Experimental data under a 5-Oe dc external field (open squares) and the fitting curves using the first-order Langevin function (dashed line) and the modified Langevin function (solid line). Inset (a) is a zooming in the temperature range of 350 to 360 K. Inset (b) is the temperature dependence of *P_2_*.

**Figure 3:**
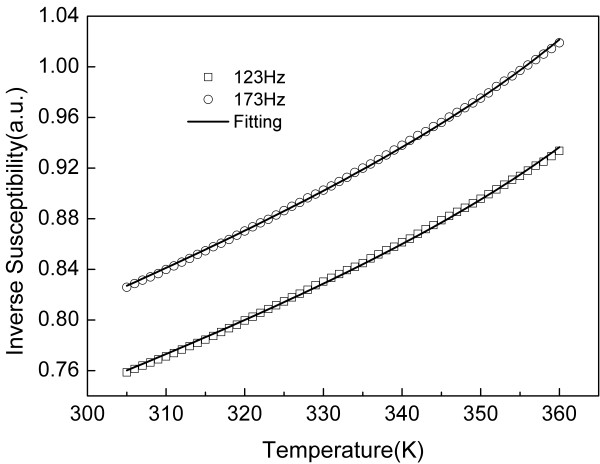
**Experimental data and the best fitting curves**. Experimental data under an ac external field (2-Oe amplitude) with frequencies set at 123 and 173 Hz (open squares and open circles, respectively) and the best fitting curves using Equation 5 (solid lines).

The experiment includes three steps: Firstly, the sample is placed inside a water bath, slowly heated up to the water's boiling point temperature, and kept there for a certain period of time. While keeping the sample holder stably hot, a weak uniform dc/ac field was applied to the MF sample. The second step is to record the inverse susceptibility (*χ*^-1 ^= *H*/*M*) versus temperature curves. The heating circuit is shut off, allowing the sample of cooling down naturally while synchronously recording both the temperature and the chord susceptibility. To avoid stray magnetic field interference, when the dc field is applied, the direction was set to invert every 0.5 s. The magnetization was evaluated according to the difference between the two measurements, before and after switching the relay. For ac susceptibility measurement, the digital phase-sensitive detection algorithm was used to calculate the amplitude of the magnetization. The last step is data processing. Regarding the data obtained from repeated experiments at different excitation fields, we took a temperature point every 0.5 K in the temperature range of the experiments, and the measured values at temperature points within a permissible measurement error range are considered to be achieved at the same temperature. Accordingly, the mean values of repeated experiments were obtained to draw the curves of inverse susceptibility versus temperature. Typical (1/*χ*) × *T *data are shown in open symbols in Figures 2 and 3. Note the superlinear behaviors of the (1/*χ*) × *T *data at the higher temperature end in both Figures 2 and 3.

## Model and discussion

### The susceptibility model for pure monomers

Considering only isolated particles (monomers) with no interparticle interaction, the MF sample's magnetization is well described by the Langevin function:

(1) $$M=\varphi M_{S}\left(\coth\left(mH/)kT\right)-kT/)mH\right),$$

where *φ *is the concentration of the nanoparticle (the number of particles per unit volume), *m *is the nanoparticle's magnetic moment, *H *is the applied field, *k *is the Boltzmann constant, and *T *is the absolute temperature. The nanoparticle's magnetic moment (*m*) is described in terms of the saturation magnetization (*M_S_*) as m = *M_s_V*, where *V *is the nanoparticle's volume. At weak enough applied fields, the sample's susceptibility *χ *= *∂M*/*∂H *approaches the chord susceptibility *χ *= *M*/*H*. Then, the inverse initial susceptibility can be written as:

(2) $$\chi^{-1}=\left(H/)\varphi \right)/)\left[M_{S}\left(\coth\left(mH/)kT\right)-kT/)mH\right)\right].$$

Equation 2 describes the (1/*χ*) × *T *data based on the first-order Langevin function. Note that the dashed line in Figure 2 is the curve fitting of the data (open symbols) using Equation 2, showing the expected linearity in the temperature range of interest. However, our experimental data (open symbols in both Figures 2 and 3) revealed a superlinear trend at the high temperature end, making the fitting procedure using Equation 2 visibly poor (see inset (a) of Figure 2). We then hypothesized that the assumption of only monomers in the MF sample in the temperature range of 305 to 360 K no longer holds.

## Discussion of the susceptibility model including dimers

The explanation of the superlinear behavior observed on the (1/*χ*) × *T *experimental data displayed in Figures 2 and 3 (open symbols), as we claim, is due to the presence of a fraction of chain-like structures within the MF sample, in addition to monomers, more likely dimers [13,14], and the temperature dependence of the dimer fraction in a critical way around T*, as discussed below.

The probability of agglomeration and disruption of suspended nanoparticles in MFs is assumed to be dependent upon the relative strength of magnetic, van de Waals, electrostatic, and steric interactions and thermal energy. The literature [15-17] describes that magnetic dipolar interaction held particles together, whereas electrostatic interaction and thermal energy work together taking nearby particles apart. It can be inferred from the experimental observations that there is a dynamic balance between the relative content of monomers and dimers within the simplest model picture of a magnetically textured MF sample. This balance will be broken when there is a change on the parameters governing the energy terms involved, and the relative content of monomers and dimers will change accordingly. As the thermal energy increases, the dimers tend to disrupt into monomers, leading to a decrease of the dimers' relative content. Inversely, within this simplest model picture, when lowering the temperature of a MF sample, the monomers tend to agglomerate into dimers, leading to a decrease of the monomers' relative content.

The already described process of the thermal-assisted dimer disruption within a MF sample [12] is related to a second-order phase transition at a critical temperature T*. Below T*, the suspended nanoparticles are found as monomers and dimers, whereas above T*, nanoparticles are essentially isolated within the MF sample while the relative content of dimers drops down critically to nearly zero around T*. Below the critical temperature T*, monomers and dimers do coexist within the MF sample. In order to describe this mixed (magnetically textured) system and the influence of the temperature upon it, we have modified Equation 1 including two contributions, namely terms due to monomers and dimers. Below T*, the probability of finding monomers (*P_1_*) and dimers (*P_2_*) within the MF sample scales with the temperature according to:

(3) $$P_{1}=T/)T^{*}\&P_{2}={\left(1-T/)T^{*}\right)}^{\beta }.$$

Considering the classical Landau's theory of second-order phase transition, *β *in Equation 3 represents the critical exponent (*β *= 0.5) as long as *P*_2 _properly describes the order parameter of the thermally assisted dimer disruption process. Thus, the modified Langevin function describing the actual magnetization of the MF sample is:

(4) $$M=\varphi P_{1}M_{S1}\left[\coth\left(m_{1}H/)kT\right)-\left(kT/)m_{1}H\right)\right]+\varphi P_{2}M_{S2}\left[\coth\left(m_{2}H/)kT\right)-\left(kT/)m_{2}H\right)\right],$$

where *m*_1 _and *m*_2 _are the magnetic moments of monomers and dimers, respectively. In Equation 4, *φP*_1 _and *φP*_2 _represent the content of monomers and dimers, respectively. For abbreviation, we write *ξ*_1 _= *m*_1_*H*/*kT *and *ξ*_2 _= *m*_1_*H*/*kT*. According to Equations 3 and 4, the inverse susceptibility describing the magnetically textured MF sample can be written as:

(5) $$\chi^{-1}=H/)\left\{\varphi P_{1}M_{S1}\left[\coth\left(\xi_{1}\right)-\left(1/)\xi_{1}\right)\right]+\varphi P_{2}M_{S2}\left[\coth\left(\xi_{2}\right)-\left(1/)\xi_{2}\right)\right]\right\}.$$

The low-field dc susceptibility data are shown in Figure 2. The solid line represents (see Figure 2) the best curve fitting of the (1/*χ*) × *T *experimental data (open squares) using Equation 5. Included here for comparison, the dashed line in Figure 2 represents the fitting of the data using Equation 2. Note that ferromagnetic resonance data (resonance line splitting) have been used to describe dimer disruption in a nickel ferrite-based ionic MF sample, providing values of T* (340 K) and *β *(0.42), the latter in reasonable agreement with the value we found in the present study [13]. Our data indicate that above T*, the suspended particles within the MF sample investigated are essentially isolated (monomers). The inset (a) of Figure 2 shows a detail of the (1/*χ*) × *T *data, emphasizing the superlinear behavior at temperatures close to the typical dimer disruption temperature (T*). The inset (b) of Figure 2 shows the temperature dependence of *P_2_*, here, describing the order parameter associated to a second-order-like phase transition.

Experiments on low-field ac susceptibility at different frequencies (123 and 173 Hz) are shown (symbols) in Figure 3. The saturation magnetization of monomers (*M*_*S*1_) and dimers (*M*_*S*2_) in the same MF sample was considered to be constant. *M*_*S*1_and *M*_*S*2_were firstly acquired by the fitting of any applied field (dc or ac) and frequency, and then both *M*_*S*1_and *M*_*S*2_were used as known parameters to fit different data sets. We found different susceptibility responses while changing the frequency of the applied field. The fittings of the experimental data (solid lines) using Equation 5 show that the critical temperature decreases from T* = 381.8 down to T* = 380.4 as the frequency of the ac excitation field increases from 123 to 173 Hz, in agreement with recently reported results [18].

### Monte Carlo simulation of second-order-like dimer-monomer transition

Because a dipolar interaction between neighboring magnetic nanoparticles favors parallel alignment of their magnetic moments, a strong interaction may induce coalescence of nanoparticles and formation of magnetically coherent clusters such as dimers. Then, monomers and dimers may coexist in the system with an intermediate concentration of nanoparticles. This model picture allows us to describe the system using the Ising model in which we consider *N_p _*identical magnetic nanoparticles disposed in one-dimensional chains. We assume that each nanoparticle can be represented by a magnetic monodomain, with a magnetic moment $M_{s}{\hat{\sigma }}_{i}$ along the direction of an external magnetic field, where *σ*_i _= ± 1, *i *= 1, 2,..., *N_p_*. Then, the total energy of the nanoparticle assembly is given by

(6) $$E=-J\sum_{i=1}^{N_{p}-1}\sigma_{i}\sigma_{i+1}-h\sum_{i=1}^{N_{p}}\sigma_{i},$$

where the sum is extended to the nearest neighbors only, *J *is the nearest neighbor exchange integral, and *h*= *M_s_H*. The equilibrium magnetic moment configuration is obtained by a MC simulation using the standard Metropolis algorithm. It is interesting to find out that for systems such as MFs in which suspended nanoparticles are found as monomers and dimers, the magnetization and susceptibility can be calculated analytically through the partition function of the system, $Z=\sum_{n}e^{-E_{n}/)kT}$, where *E_n _*is the energy of the system in the *n*th configuration. For instance, we can use the following four configurations in order to describe dimers |*σ*_1_*σ*_2_>=|++>,|+->,|-+>,|-->.

**Figure 4:**
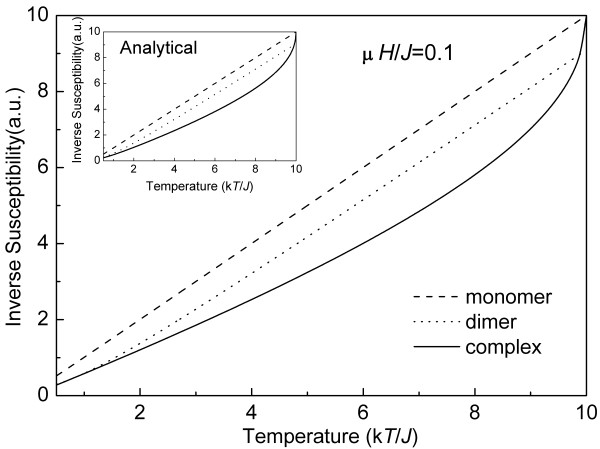
**Inverse susceptibility of monomers, dimers, and their complex**. Inverse susceptibility of monomers (dashed lines), dimers (dot-dashed lines), and their complex (solid lines) as a function of temperature for *μH*/*J *= 0.1, obtained by MC simulation. Inset presents the correspondent analytical solution.

Temperature-dependent disruption process of dimers, i.e., variation on the number of dimers (*N*_d_) in MFs, can be modeled by a parameter *γ*, which is defined as $\gamma =N_{\text{d}}/\left(N_{\text{m}}+N_{\text{d}}\right)=\sqrt{1-\left(T-T_{\text{i}}\right)/)\left(T_{\text{f}}-T_{\text{i}}\right)}/)2$, where *N*_m _is the number of monomers, *N_p _*= *N_m_*+2*N_d_*, and *T*_i _(*T*_f_) is the initial (final) temperature of the simulation. Notice that *γ *describes the system in a wide range of configurations in regard to the disruption process, for instance, from a MF with the same number of monomers and dimers (*N_d _*= *N_m_*) at *T *= *T_i _*to a configuration in which dimers are completely disrupted (*N_d _*= 0) at *T *= *T_f_*. Then, the system's susceptibility consisting of monomers and dimers (complex system) is given by

(7) $$\chi =N_{\text{p}}\left[\left(1-\gamma \right)\chi_{\text{m}}+\gamma \chi_{\text{d}}\right]/)\left(1+\gamma \right),$$

where *χ*_m _(*χ*_d_) is the susceptibility of the monomers (dimers). Figure 3 shows the temperature dependence of the inverse susceptibility obtained by MC simulation for monomers (dashed line), dimers (dash-dotted line), and their complex (solid line) at *μH*/*J *= 0.1, *kT_i_*/*J *= 0.5, and *kT_f_*/*J *= 10. Notice that the inverse susceptibility of monomers is larger than that of dimers, and both of them show almost linear dependence on temperature. However, an unusual superlinear deviation is observed in the monomer-dimer complex curve. From Equation 7, one sees that *χ *mainly depends upon *χ_m_, χ_d_*, and *γ*. Therefore, the variation of *γ *with temperature, i.e., the change of the number of dimers in a MF sample, results in the deviation of the susceptibility of MFs from linearity. Finally, the analytical solution of the monomer-dimer complex system, as shown in inset of Figure 4, gives a further strong support for this model picture.

### The pure monomer scenario above T*

**Figure 5:**
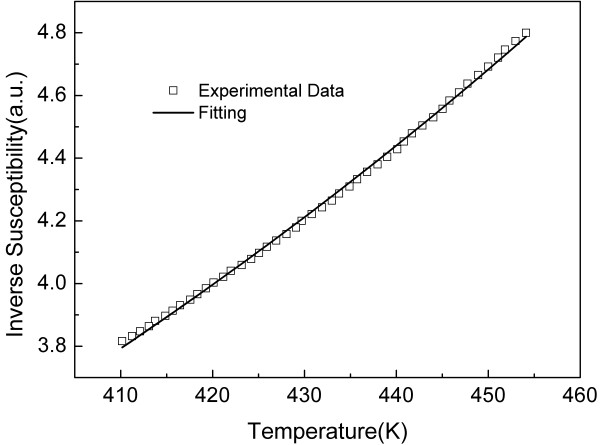
Experimental data under ac external field (2 Oe and 83 Hz) above T* (open squares). The solid line represents the best curve fitting using Equation 8.

Experimental investigation of the inverse susceptibility versus temperature was also performed in the temperature range above T*. In order to prevent volatilization of the MF solvent (mineral oil), the highest temperature achieved in our experiment was restricted to 455 K. According to the analysis presented above, all dimers are assumed to be disrupted into monomers when the temperature of the MF sample is set above the critical temperature T*. This means that above T*, the relative content of dimers tends to be 0% whereas the relative content of monomers tends to be 100%. The inverse susceptibility-temperature curve in the temperature range of 410 to 455 K is plotted in Figure 5. The experimental data shown in Figure 5 were fitted by the monomer model, herein described by:

(8) $$\chi^{-1}=H/)\left[\varphi M_{S}\left(\coth\left(mH/)kT\right)-kT/)mH\right)+A\right].$$

Note that *A *in Equation 8 means just a scale factor for the experimental data. The solid line in Figure 5 has an excellent agreement with the experimental data and presents the best curve fitting achieved using Equation 8. Furthermore, the monomer's saturation magnetization (*M_S_*) obtained from the data recorded in the temperature range above T* (from 410 to 455 K) is very much close to the value found from the data recorded in the temperature range below T* (from 305 to 360 K). Although fitting of the data presented in Figure 5 (open symbols) can be performed using the dimer-monomer model, represented by Equation 5, there is no agreement between the *M*_*S*1_obtained from below and above T*. This indicates that dimers can be assumed to be totally disrupted into monomers when the temperature is increased above T*, and the critical temperature T* can be actually used to describe a MF sample.

## Conclusion

In conclusion, the usual linear temperature dependence of the inverse initial susceptibility (dc or ac) of MFs at lower temperatures is found whereas at higher temperatures, an upward deviation of linearity is observed. This superlinear behavior is attributed to a thermal-assisted disruption of dimers into monomers which is described by the classical Landau's approach for second-order phase transitions. The experimental observations in the temperature ranges below and above the critical temperature T* are well fitted by the model picture proposed here, in which an extended Langevin function including the thermal criticality of the MF system is adopted below T*. Our findings are strongly supported by both MC simulation and analytic analysis.

## Competing interests

The authors declare that they have no competing interests.

## Authors' contributions

WL conceived the project. PCM contributed the magnetization theory and model; WL and JZ designed the experimental system; and JZ and QX performed the experiments. LOM and FQ performed the Monte Carlo simulation. All authors wrote the manuscript, discussed the results, and commented on the manuscript. All authors read and approved the final manuscript.
